# CReM: chemically reasonable mutations framework for structure generation

**DOI:** 10.1186/s13321-020-00431-w

**Published:** 2020-04-22

**Authors:** Pavel Polishchuk

**Affiliations:** Institute of Molecular and Translational Medicine, Faculty of Medicine and Dentistry, Palacky University and University Hospital in Olomouc, Hnevotinska 5, 77900 Olomouc, Czech Republic

**Keywords:** De novo structure generation, De novo design, Matched molecular pairs

## Abstract

Structure generators are widely used in de novo design studies and their performance substantially influences an outcome. Approaches based on the deep learning models and conventional atom-based approaches may result in invalid structures and fail to address their synthetic feasibility issues. On the other hand, conventional reaction-based approaches result in synthetically feasible compounds but novelty and diversity of generated compounds may be limited. Fragment-based approaches can provide both better novelty and diversity of generated compounds but the issue of synthetic complexity of generated structure was not explicitly addressed before. Here we developed a new framework of fragment-based structure generation that, by design, results in the chemically valid structures and provides flexible control over diversity, novelty, synthetic complexity and chemotypes of generated compounds. The framework was implemented as an open-source Python module and can be used to create custom workflows for the exploration of chemical space.

## Introduction

The drug-like chemical space is vastly enormous—its size estimates in ~ 10^33^ compounds [1]. In the nearest future, it will be impossible to enumerate this space or perform any kind of exhaustive search. Therefore, methods and strategies to explore this space effectively attract vivid research interest. One of the popular strategies is de novo design—model-driven generation of new chemical structures with promising predicted properties [2, 3]. Two major strategies of structure generation exist: (i) iterative generation of structures to fit model predictions and (ii) generation of structures having a desirable set of properties directly by machine learning (ML) models (e.g. inverse QSAR or generative neural networks).

The first strategy is widely used and many studies describe different implementation schemes [4–9]. The general workflow includes: (i) generation or selection of initial structures, (ii) evaluation of generated structures by the model(s) (QSAR, docking, pharmacophores, etc.), (iii) selection of the most promising candidates, (iv) generation of new structures based on the selected ones and return to the step (ii). This procedure is repeated until compounds with desirable properties are generated. Structure generation and property estimation steps are separated in this case. So one can use any combination of structure generation approaches and in silico models to predict the properties of compounds. We can divide the conventional approaches into three groups: atom-based, fragment-based and reaction-based structure generators, each having their advantages and issues (Table 1).

**Table 1:** Features of structure generation algorithms

	Atom-based	Fragment-based	Reaction-based
Exhaustiveness of chemical space search	+++ Suitable for systematic exploration of local chemical space	++ Variable, controlled by the size and diversity of fragments	+ Depends on diversity of a reactant library and a list of annotated reaction rules
Structure novelty	+++ Many steps to achieve high novelty	++	+
Structure diversity	+++ Many steps to achieve high diversity	++	+
Chemically valid structures	–	–/+	+++
Synthetically feasible structures	–	–/+	+++
Efficiency due to possible combinatorial explosion	–	++	+++

Atom-based approaches represent “ab initio” methods among structure generators and use simple rules like “add/remove/replace atom/bond” to modify input structures and generate new ones [10]. Theoretically, it should be possible to generate every possible structure using these rules, which can result in high novelty and diversity of enumerated structures. However, a lot of generation steps will be required resulting in a combinatorial explosion. Therefore, atom-based approaches suit better for the systematic exploration of a local chemical space. Chemical validity should be additionally controlled during structure generation to avoid erroneous structural changes. However, the main issue of atom-based approaches is synthetic feasibility, which cannot be controlled over the course of generation and may result in synthetically less accessible structures. To the best of our knowledge, there is only one implementation of the atom-based generator—Molpher [10].

Reaction-based approaches generate new compounds by applying the rules from a list of encoded chemical transformations to a library of reactants [7]. As it is understandable intuitively, reaction-based approaches produce the higher novelty and diversity in just a few generation steps compared to atom-based approaches, which may require much more steps to achieve the same goal. Reaction-based approaches make large changes in structure during compounds generation and, therefore, seem more suitable for the rough exploration of chemical space. With a comprehensive reactant library it should be also possible to enumerate the close analogs of a reference compound for a local exploration of chemical space. Synthetic feasibility of generated compounds and an available synthetic route are the main advantages of reaction-based approaches. Applicability of this kind of approaches was demonstrated in several studies [7, 11–13]. Nevertheless, the limited number of rules (mainly only coupling reactions are considered) and the limited size of reactant libraries may restrain these algorithms from exploring larger chemical space (therefore losing novelty and diversity of generated compounds).

Fragment-based approaches are in-between of atom-based and reaction-based ones [5, 9, 14]. They replace or add whole groups of atoms at once. The set of initial fragments directly determines the novelty and diversity of generated compounds. So we expect that fragment-based approaches will outperform reaction-based ones (in terms of diversity and novelty) as it seems easier to collect a diverse library of fragments than a diverse library of reactants. One can also control the exhaustiveness of chemical space exploration by varying the size of fragments. However, an accessible chemical space is smaller than for atom-based approaches. It seems to be easier to control chemical validity of enumerated structures in case of fragments, but synthetic feasibility still presents an issue because linking of synthetically feasible fragments may result in synthetically infeasible molecules. Therefore, fragment linking should take into account the chemical context of coupled fragments. Recently Liu et al. published an approach where they took into account types of atoms to which fragments can attach [15]. Their strategy resulted in generation of chemically valid structures but the authors did not study synthetic feasibility of generated structures and the context of one atom may be insufficient to guarantee generation of synthetically feasible molecules. Moreover, the authors provided only an open-source implementation of a fragmentation procedure, but not the implementation of structure enumeration. Many other fragment-based approaches exist but the majority of them are not publicly available as software solutions [6, 8, 16, 17]. The FOG algorithm was implemented as a standalone tool and distributed with OpenGrowth software for molecule growing inside the protein pocket [9] but it can only grow molecules that may limit search space.

The interest in the second strategy of structure enumeration rekindled recently due to the advances in deep learning and generative models [18–24]. Compound structures can be generated in an unsupervised or supervised manner. In unsupervised approaches ML models (usually recurrent neural networks) are trained on structures of known compounds (usually represented by SMILES) and stochastically sample output structures. To make generation more focused a model trained on a large diverse set of compounds can be post-trained on a small subset of compounds active against a particular target. This creates a bias in the model to make it generate compounds more similar to the active ones [24]. In supervised approaches a model is optimized to find a combination of descriptors that correspond to active compounds and then structures of compounds are reconstructed from this descriptors set (so-called “inverse QSAR” task). The conventional descriptors proved to be very difficult to reconstruct structures. Several approaches were developed in the past but gained low popularity due to many restrictions and limitations [25–30]. The recent advances in deep learning allowed to generate a latent representation of input compounds, find an optimal set of the latent variables associated with desired property values and sample structures from this latent subspace of variables [31]. Nevertheless, no guarantee exists that the found optimal combinations of latent variables correspond to valid chemical structures. The percentage of valid structures generated by deep learning models can vary a lot depending on the deep learning model architecture: from almost 100 to 4% [32]. Moreover, the currently developed approaches have no control over the synthetic feasibility of generated structures which is usually estimated after compounds generation [31].

Despite the recent successes in generative deep neural network models, fragment-based approaches seem an attractive alternative because they provide high flexibility of exploration of chemical space with reasonable efforts and can be coupled with any modeling approach. They may also address the synthetic accessibility issue but this was not investigated so far. At the moment there is no open-source software that implements different modes of fragment manipulation (mutate, grow and link) and provides a convenient program interface for integration with third-party software to develop custom search workflows. In this study, we developed a framework of fragment-based structure enumeration that provides all basic functions for manipulating fragments and is easy to integrate with third-party software. The approach is based on the determination of interchangeable fragments from databases of known compounds to perform chemically reasonably mutations (CReM) of input structures. It generates chemically valid structures by design and allows to indirectly control the synthetic feasibility of enumerated compounds as well as their chemotypes.

## Implementation

**Fig. 1:**
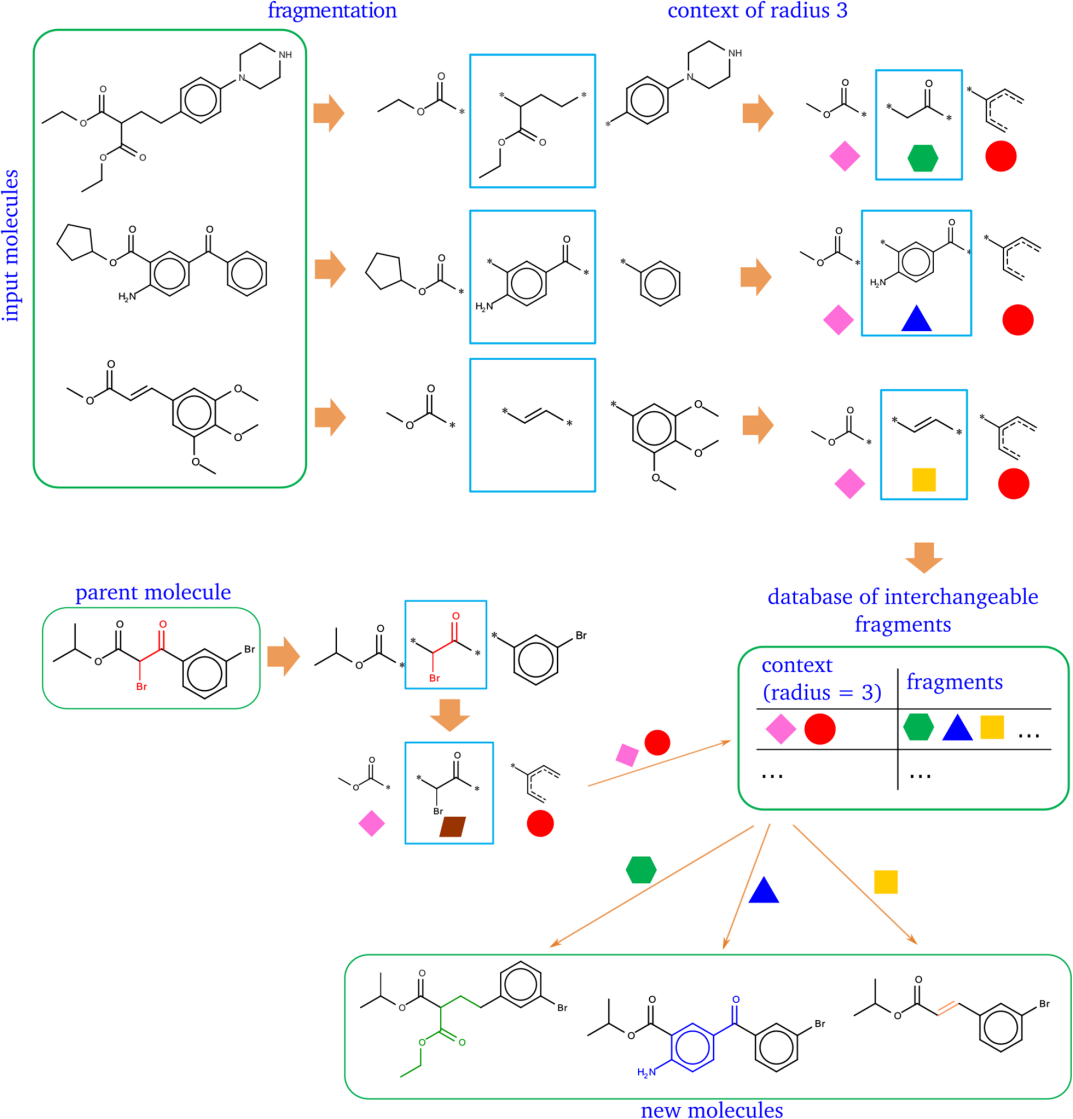
Generation of a database of interchangeable fragments and new molecules

The idea of interchangeable fragments—the core of the developed approach—is directly related to the matched molecular pairs approach considering their local context [33]. Interchangeable fragments are fragments that occur in the same local chemical context in structures of known compounds (Fig. 1). Atoms within a particular radius around attachment points of a fragment represent this local chemical context. We replace one fragment by another having the same chemical context, which should result in a chemically valid and feasible structure. Thus, by design, the chemical validity of generated structures is guaranteed. Intuitively, it can be also expected that the generated compounds are synthetically feasible.

**Fig. 2:**
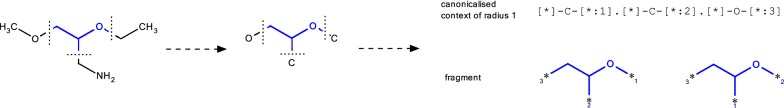
Canonicalization of attachment point numbers in contexts and fragments

Generation of a database of interchangeable fragments is a two-step procedure. On the first step, structures of known compounds are exhaustively fragmented by cutting up to 4 non-cyclic single bonds between two heavy atoms using RDKit implementation of the matched molecular pairs algorithm suggested by Husain and Rea [34]. Hydrogens are cut separately. On the second step, a context of a given radius is determined for attachment points of each fragment and encoded in a SMILES string. This SMILES string is canonicalized to get both a canonical numbering of attachment points and canonical SMILES representation of a context. Attachment points in a corresponding fragment are renumbered correspondingly. SMILES representation of a context of a given radius and an associated fragment are stored in a database table as a key-value pair for a subsequent search of interchangeable fragments (values) having an identical context (key) (Fig. 1). If a context of two or more attachment points is identical, all possible permutations of these attachment points in a corresponding fragment are performed. The numbers of attachment points in the context are not changed because this will result in identical SMILES representation. Fragments with alternative attachment point numbering are stored individually as key-value pairs (context, in this case, is the same). This situation is illustrated in Fig. 2. The central fragment has three attachment points. Two of them with numbers 1 and 2 have the identical context of radius 1 (–CH_2_-group). Therefore, all possible permutations of attachment points in a fragment which are consistent with the attachment point numbering in a context are enumerated. In this case, 1 and 2 are swapped and both fragments with different numbering are stored in a database. This is done to be able to make all possible replacements if some attachment points are equivalent.

To replace a fragment in a molecule its context of a given radius is determined and canonically encoded. The given SMILES string of a context is searched in a fragment database and fragments with the same context are retrieved and used for fragment replacement (Fig. 1).

**Fig. 3:**
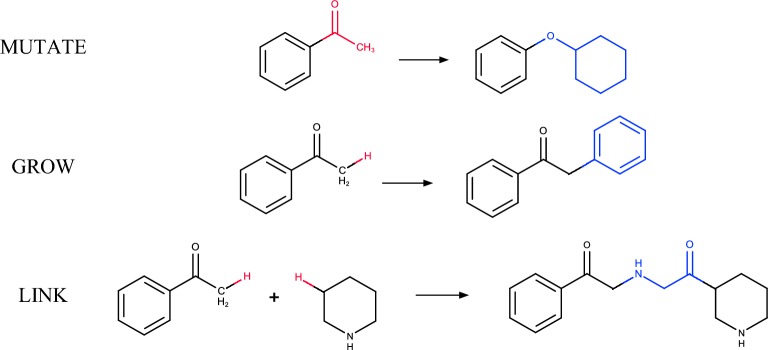
Structure generation modes

We implemented three modes of structure generation: MUTATE, GROW and LINK (Fig. 3). Mutate is a replacement of an arbitrarily chosen fragment with another one. GROW is a special case of a MUTATE operation—replacement of a hydrogen with another fragment. LINK is a replacement of hydrogen atoms in two molecules to link them by an appropriate fragment.

Several tuning parameters are available:

1. Structures of the input compounds used to create a database of interchangeable fragments.
  Management of the content of the input compound database used for fragmentation gives indirect control over enumerated structures and provides additional flexibility. The selection of synthetically feasible input compounds may improve synthetic feasibility of generated compounds. At the same time pre-selection of compounds for fragment library enumeration may reduce diversity and novelty of generated structures.

2. Radius of a considered molecular context.
  Increasing the radius of a considered molecular context will decrease the appearance of new chemotypes in enumerated compounds and make replacements more conservative.

3. Frequency of occurrence of interchangeable fragments in the input database.
  Similarly to the synthetic accessibility score suggested by Ertl & Schuffenhauer [35] it can be supposed that replacement with more frequently occurred fragments will lead to more synthetically feasible compounds. This will also reduce the number of replacements and increase search speed.

4. Size of fragments which will replace each other.
  The size of replaceable fragments can control exhaustiveness of chemical space exploration by increasing or decreasing search steps and depends on the goal of a particular study, thus, it will not be specifically investigated here. Lead optimization studies may require small steps to explore local chemical space around a parent compound, whereas lead generation may require large steps in the beginning to quickly and coarsely explore larger chemical space and smaller steps in the end to finely tune generated structures.

5. Maximum number of randomly chosen replacing fragments.
  Limiting the maximum number of replacements can speed up the exploration of a chemical space as generated fragment databases can be very large and making all possible replacements can be costly. We will not specifically investigate this parameter in this study.

6. Protection of selected atoms from modification or modification of only selected atoms.
  This functionality can be useful for property/activity optimization studies to protect scaffold or pharmacophore features from changes or to modify molecules only at specific positions. We will not investigate this option here.

With all these options the developed approach possesses great flexibility and control over generated structures.

## Methods

There are still no commonly used criteria to measure the performance of structure generators and the quality of virtually enumerated libraries. We will use several cases to explore the effect of selected tuning parameters on generated structures.

First, we will simulate local exploration of chemical space around parent compounds taken from DrugBank. We will apply the MUTATE operation once for each parent compound. This will correspond to a single step in chemical space. In this way, we will explore the dependence of novelty, diversity and synthetic complexity of generated compounds on CReM tuning parameters: content of a fragment database, context radius and frequency of occurrence of fragment-context pairs. The novelty of generated compounds will be calculated as a mean Tanimoto distance to a parent compound based on 2048-bit Morgan fingerprints of radius 2 calculated in RDKit. This will show how dissimilar the generated compounds are from a parent compound (the higher the score, the better). Additionally, we will estimate novelty by counting the percentage of generated compounds, which are not available in the fragmented ChEMBL compounds. We will refer to this as ChEMBL novelty. The diversity of generated compounds will be calculated as a mean Tanimoto distance based on 2048-bit Morgan fingerprints of radius 2 between all pairs of generated compounds. In case if the number of generated compounds is large a random subset of 1000 compounds will be used to estimate the diversity of generated structures. This procedure will be repeated five times to estimate robustness of the obtained value. Diversity will show how intrinsically diverse the sets of generated compounds are (the higher the score, the better). It is expected that novelty and diversity will be highly correlated because novelty (as defined above) can be interpreted as diversity relative to a reference compound. Synthetic complexity is predicted by the model developed by Coley et al. (SCScore) [36] and by the model developed by Ertl and Schuffenhauer (SAScore) [35]. SCScore values lie within the range from 1 to 5, where synthetically feasible compounds have score 1, whereas synthetically complex compounds are closer to score 5 (the lower score, the better). SAScore values are within the range from 1 to 10, where synthetically feasible compounds have score 1 and synthetically complex compounds are closer to score 10 (the lower score, the better).

To investigate the hypothesis that limiting synthetic complexity in fragmented structures improves synthetic accessibility of generated compounds we created fragment libraries from all ChEMBL compounds with SCScore below a specified threshold (2, 2.5, 3, 3.5). The fixed context radius 3 was chosen as a reference because at this radius most functional groups are distinguishable and, therefore, structural replacements are reasonably specific. At the smaller radius some functional groups cannot be distinguished, e.g. N-substituted amide (*–N–C(=O)) and amino (*–N–C–C) groups. Larger radius, on the other hand, would result in too specific replacements because it would be able to distinguish amides differently substituted at α-carbon atom.

Second, we will perform iterative stochastic exploration of chemical space starting from benzene molecule. This study should confirm that new structural motifs of the size equal to the considered context radius or less cannot be generated. Here size means the longest distance between two atoms in a fragment, where the distance is the shortest path between two atoms. By increasing the radius one can make a generation of structures more conservative relative to chemotypes of compounds used for generation of a fragment database. This can be useful in a scenario, where compounds with undesirable patterns are removed from the input database and a fragment database is generated with a reasonably large radius to avoid generation of compounds with these undesirable motifs (chemotypes). For this case study we will use a database of fragments generated from compounds not containing PAINS patterns [37]. The PAINS patterns were detected by rules implemented in RDKit. We will also investigate the physicochemical properties of generated compounds and Bemis–Murcko scaffolds to characterize chemical space of enumerated compounds and demonstrate the ability to generate novel scaffolds. Bemis–Murcko scaffolds and physicochemical properties were also calculated by RDKit scripts.

Recently several papers were published to address the issue of evaluation of structure generation approaches [38, 39]. As the third case study we will use the Guacamol benchmarks [38]. There are two types of benchmarks: distribution learning and goal-directed. The former estimates the ability of a structure generator to reproduce within generated compounds the distribution similar to reference chemical space. The latter is a set of 20 tasks which goal is to rediscover known drugs, generate compounds similar to the reference ones, perform multi-objective optimization of properties of known drugs, or make scaffold hopping. This should demonstrate the general applicability of the CReM approach.

## Results and discussion

### Fragment databases generation

ChEMBL database (version 22) was used as a source of structures for the databases of interchangeable fragments. 1,557,992 distinct structures containing only organic elements (C, N, O, S, P, F, Cl, Br, I, B) remained after the curation procedure. The curation was performed using the previously developed protocol [40] which includes Chemaxon Stardardizer and Checker [41] and RDKit [42] sanitization checks. To estimate the context radius’ effect on the generated compounds we generated two databases with context radius from 1 to 5. The first database was generated from all ChEMBL compounds. The second one was generated from compounds not containing PAINS patterns (1,464,907 compounds). The number of distinct combinations of contexts and fragments grew linearly with context radius increase (Table 2). Several databases were generated from all ChEMBL compounds having limited SCScore values. The number of compounds and the resulting fragment and context pairs substantially decreased with lowering the SCScore threshold (Table 3).

**Table 2:** The number of distinct fragments and corresponding contexts in databases generated from the whole ChEMBL data set and its PAINS-less subset

Radius	ChEMBL	PAINS-less ChEMBL
1	35,833,160	34,240,810
2	41,676,473	39,800,734
3	51,730,960	49,403,630
4	62,821,316	59,971,431
5	74,168,168	70,717,996

**Table 3:** Statistics of the initial ChEMBL data set and filtered ones by SCScore values and the number of the resulting distinct fragment and context pairs

Data set	Number of compounds	Number of distinct fragments & contexts of radius 3
ChEMBL	1,557,992	51,730,960
SCScore <= 3.5	552,162	20,514,883
SCScore <= 3	284,461	10,661,179
SCScore <= 2.5	111,365	4,091,634
SCScore <= 2	27,916	951,993

### Case study 1: Local chemical space exploration

#### Parent compounds selection

DrugBank compounds were selected as a source of the parent compounds for simulation of a local exploration of chemical space. 6002 compounds remained after the curation of the whole database using the protocol mentioned above. To select compounds of different complexity we ranked them according to their SCScore values and each twelfth compound was selected to create a subset of 500 parent compounds.

The MUTATE operation was applied to the selected 500 compounds. We set the minimum size of the replaced fragment to 0 to enable the replacement of hydrogens. The maximum size of the replaced fragment was set to 10 heavy atoms. The size of a replacing fragment could be smaller or larger than the replaced fragment by at most two heavy atoms.

#### Influence of a context radius on generated compound sets

**Fig. 4:**
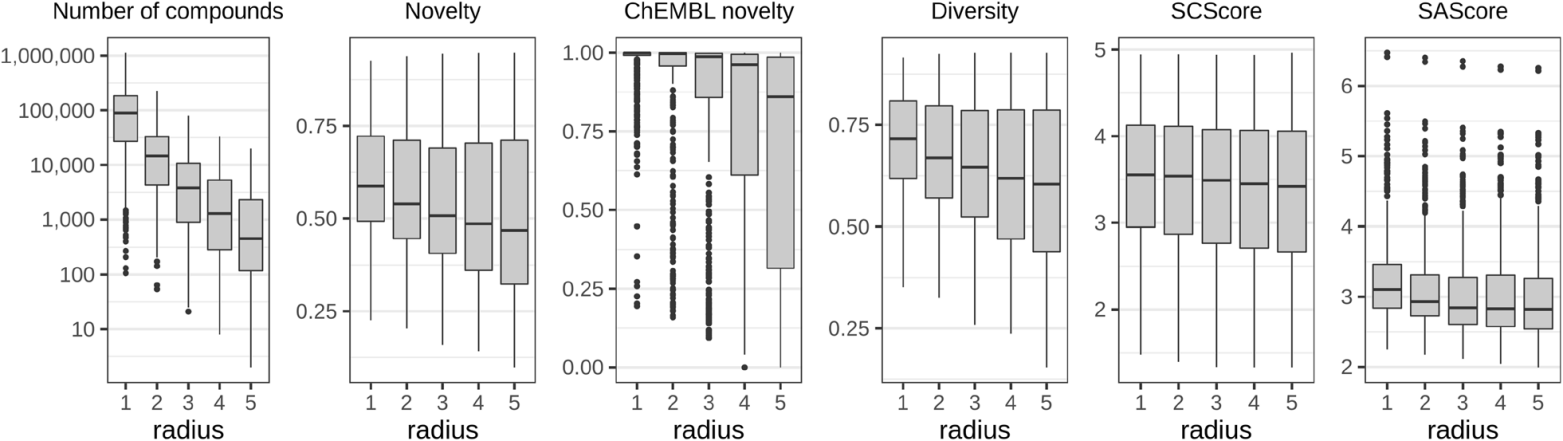
Distributions of the number of generated compounds and average novelty, diversity and synthetic complexity based on 500 compound sets generated from DrugBank compounds using whole ChEMBL fragment database with different context radius

The database of fragments generated from the whole ChEMBL data set was used to study the influence of a context radius on generated sets of compounds. Starting from the selected 500 DrugBank compounds the corresponding number of data sets was generated; after that average novelty, diversity and synthetic complexity were calculated for each data set. As we expected the number of generated compounds substantially dropped with the increase of context radius because a smaller number of matching context-fragment pairs were found in a fragment database. Average novelty and diversity decreased more smoothly and generated data sets still covered a large range of these values. ChEMBL novelty dropped steeper, but still the majority of generated structures did not occur in ChEMBL. Synthetic complexity seemed to be the least affected, but it also decreased with the increase of context radius (Fig. 4).

**Fig. 5:**
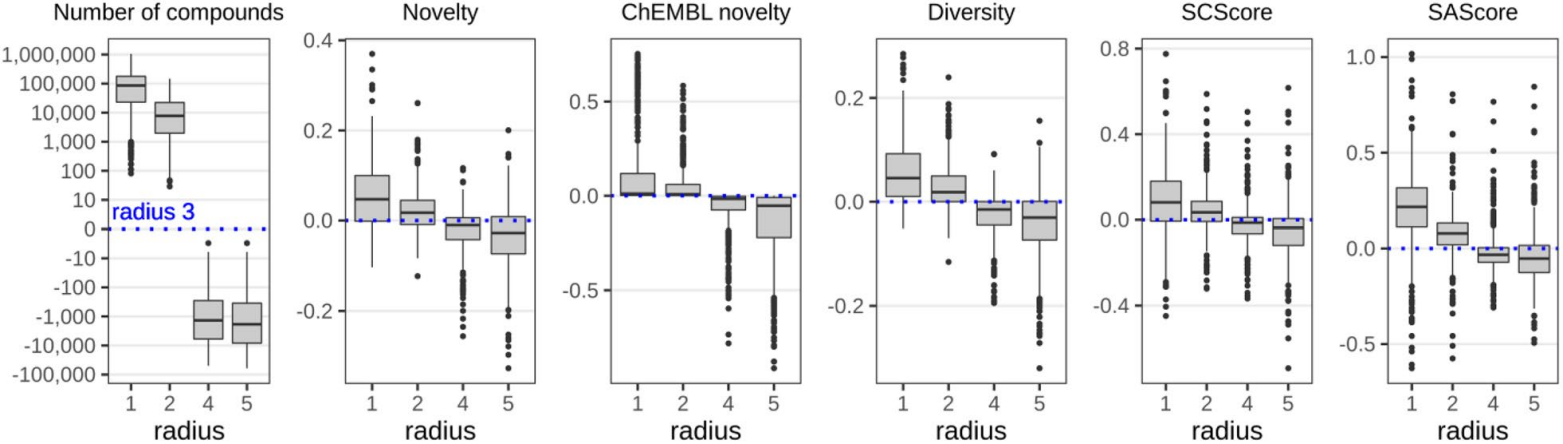
Distribution of differences in the number of compounds and average novelty, diversity and synthetic complexity between data sets generated using context radius of 3 (reference) and others. Positive values demonstrate that parameter values of a data set are greater than for data sets generated at radius 3 and vice versa

The general distributions of data set parameters depicted in Fig. 4 struggle to give a complete picture of the properties of the generated data sets. Therefore, we plotted differences to emphasize the changes of parameters depending on a context radius for each parent compound. We selected data sets generated at the context radius 3 as reference points and calculated differences between corresponding parameters of data sets generated at another context radius for the same parent compound (Fig. 5). The results revealed the same trend in a more pronounced way. Despite the substantial drop in the number of generated compounds up to 1 million, novelty, diversity and synthetic complexity were not decreased much with increasing the context radius from 1 to 3. An increase in the context radius to 4 or 5 resulted in a continuation of a decrease of the number of generated compounds on up to 100,000 and a slight decrease of novelty, diversity and synthetic complexity.

#### Control over synthetic complexity

We examined two possible strategies to reduce the synthetic complexity of generated compounds. The first one was based on the idea that constructing structures from frequently occurring fragments results in more synthetically feasible compounds. We used the whole ChEMBL fragment database and limited replacements by the fragment occurrence in the database: no limits or minimum 10, 100 or 1000 occurrences. The second strategy was based on the fragment databases generated from more synthetically accessible compounds according to SCScore [36]. No restrictions on the fragment occurrence were applied in this case. The same 500 DrugBank compounds were used as parent structures and the corresponding number of compound sets were generated using each of these strategies.

Table 4 shows the number of times each generation strategy resulted in a data set with the best average synthetic complexity, novelty, and diversity. The strategy, which used whole ChEMBL fragment database with no restriction, (ChEMBL & 0) resulted in the highest novelty and diversity of enumerated data sets in about 70% of cases, whereas the lowest average synthetic complexity was observed in only 13 (SCScore) or 29 (SAScore) cases (2.6% and 5.8%, respectively). Average ChEMBL novelty was the highest in all 500 cases.

**Table 4:** Comparison of different generation strategies

Fragment database & fragment occurrence	Wins (by lower mean SAScore)	Wins (by lower mean SCScore)	Wins (by higher mean novelty)	Wins (by higher mean diversity)	Mean/median number of compounds
ChEMBL & 0	29	13	357	347	7812/3800
ChEMBL & 10	44	5	2	47	695/323
ChEMBL & 100	76	17	1	7	98/66
ChEMBL & 1000	229	169	0	4	19/15
SCScore ≤ 3.5 & 0	11	16	55	61	5331/2540
SCScore ≤ 3 & 0	12	19	29	24	4031/1898
SCScore ≤ 2.5 & 0	30	56	16	9	2473/1122
SCScore ≤ 2 & 0	69	205	40	1	1040/430
Total number of compounds:	500	500	500	500	

Using frequently occurred fragments for replacement resulted in an increased number of generated data sets with lower synthetic complexity. As expected, this had the most pronounced effect on SAScore with up to 45.8% winning cases for the strategy used fragments occurred at least 1000 times in the whole ChEMBL database (ChEMBL & 1000). The average SCScore of generated data sets was less affected. But ChEMBL & 1000 could result in 33.8% of cases with the lowest average SCScore values. This was the second-best strategy with respect to SCScore. The corresponding top strategy with the greatest number of wins (205 cases or 41%) was based on the fragment database generated from compounds with SCScore ≤ 2. It should be noted that the limitation of fragment replacements based on fragment occurrence substantially decreased the number of output compounds. The average number of generated compounds was 15 in the case of ChEMBL & 1000 whereas the strategy used the fragment database generated from compounds with SCScore ≤ 2 resulted in 430 compounds on average. The latter strategy outperformed all strategies based on fragment occurrence limitation by the number of generated compounds.

**Fig. 6:**
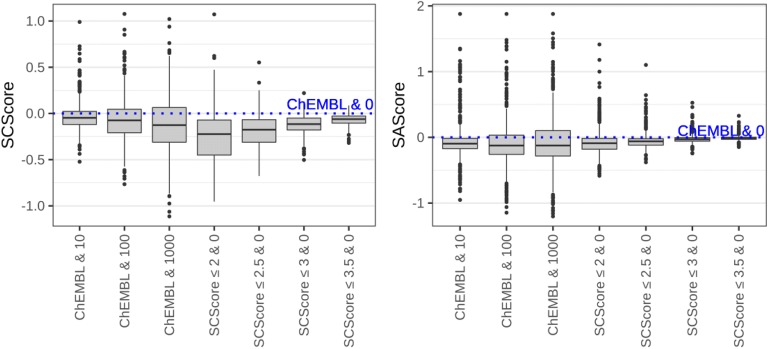
Changes in average synthetic complexity of data sets generated from 500 DrugBank compounds used different restricted strategies relatively to an unrestricted generation used the whole ChEMBL fragment database. The occurrence of a replacing fragment in a database is given after an ampersand symbol

Synthetic complexity estimated by SCScore reduced more pronounced in cases where fragment databases generated from more synthetically accessible compounds were used (Fig. 6). In the case of the fragment database created from compounds with SCScore ≤ 2 the average decrease was 0.26, which is comparable to the value 0.25 imposed by the authors of SCScore as an objective during optimization to separate reactants and products. Therefore, it might be expected that compounds having SCScore value lower by 0.25 would require one less step to be synthesized. The SAScore was also lowered in this case. This demonstrates that the selection of synthetically feasible compounds for generation of fragment databases can reduce the synthetic complexity of output structures.

### Case study 2: Stochastic exploration of chemical space

#### Control over chemotypes of generated compounds

We simulated an unrestricted iterative stochastic exploration of a chemical space starting from a benzene molecule to demonstrate the ability of CReM approach to avoid generation of structural motifs if their size is less than the chosen context radius. On each iteration, the MUTATE operation was applied to input compounds. The following setup was chosen for this study:

- the size of the replaced fragment was varied from 0 to 8 heavy atoms;
- the relative size of the replaced fragment (the number of heavy atoms in a fragment relative to the whole molecule) should not be greater than 0.3 (to avoid large structural changes in the beginning);
- the size of a replacing fragment should not differ on more than one heavy atom from the size of a replaced fragment to make small steps in chemical space;
- the maximum number of replacements was limited to 25000 randomly chosen ones to speed up the simulation.

Generated compounds with molecular mass greater than 500 were discarded. The remaining compounds were ordered by molecular mass and split into 5 bins and one compound was randomly selected from each bin. The selected five compounds passed to the next iteration. Totally 100 iterations were executed.

**Fig. 7:**
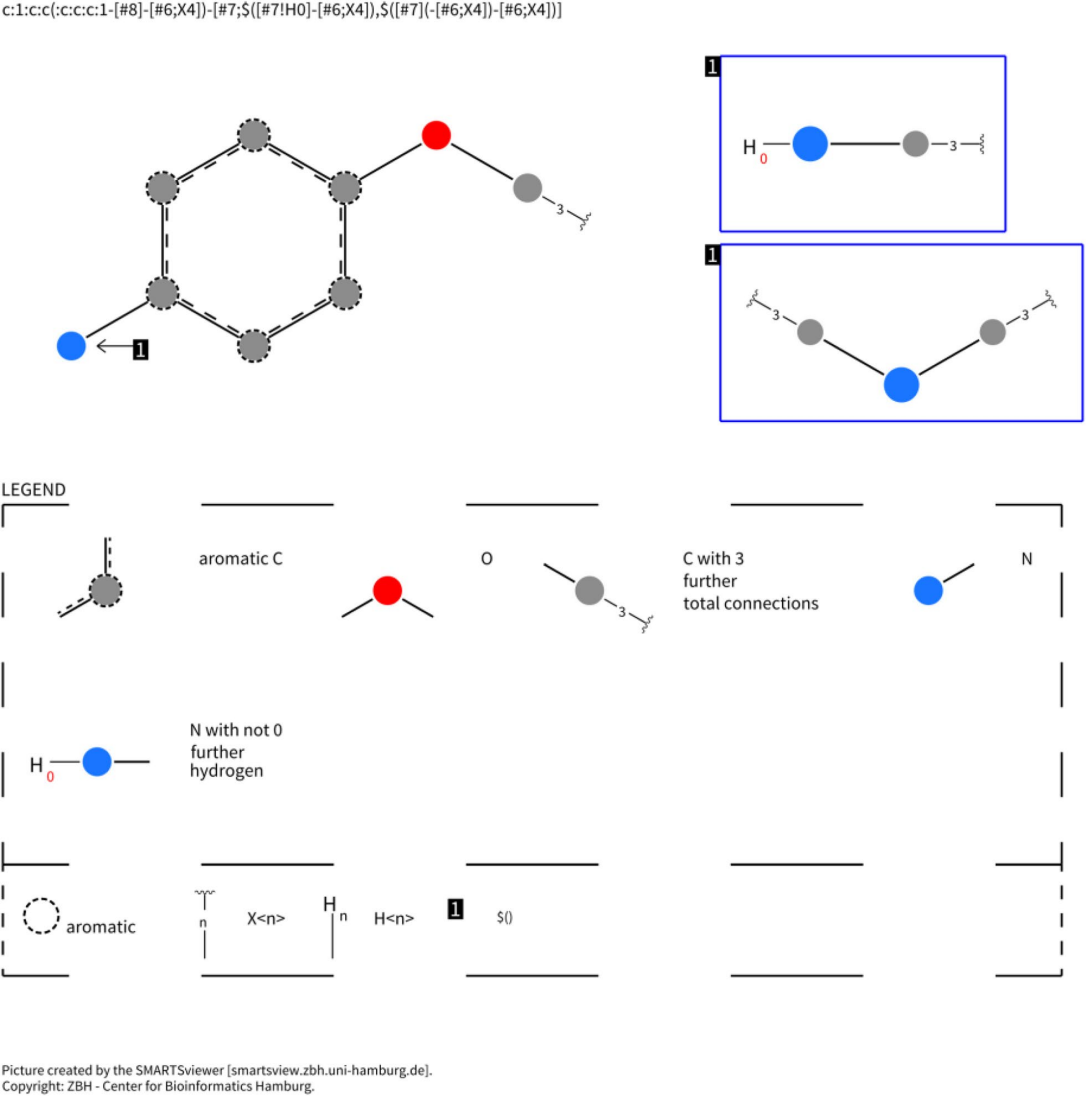
The structure of PAINS anil_di_alk_C(246). The image was produced with SMARTSviewer [43]

This procedure was run once per each context radius from 1 to 5 using the PAINS-less fragment database. Then compounds generated on all iterations of each run were collected and examined to contain PAINS fragments. As expected, the number of emerged PAINS patterns decreased with the increase of context radius: 102 distinct PAINS patterns for radius 1 were detected, 52 for radius 2, 28 for radius 3, 26 for radius 44 and 1 for radius 5. The last one is dialkyl aniline moiety having a methoxy substituent at the *para*-position (Fig. 7). The longest distance in this pattern equals to 6 bonds between the nitrogen atom and the methoxy carbon atom, therefore the context of radius 5 could not fully cover it and the motif was reconstructed during structural mutations. Only one PAINS pattern was generated due to the stochastic nature of the simulation but other PAINS fragments having the size greater than 5 may appear in structures generated with context radius 5. However, no smaller patterns were detected. This demonstrated that one can implicitly control chemotypes of generated compounds by an increase of context radius. The full list of determined PAINS patterns is provided in Additional file 1.

#### Murcko scaffold analysis

**Fig. 8:**
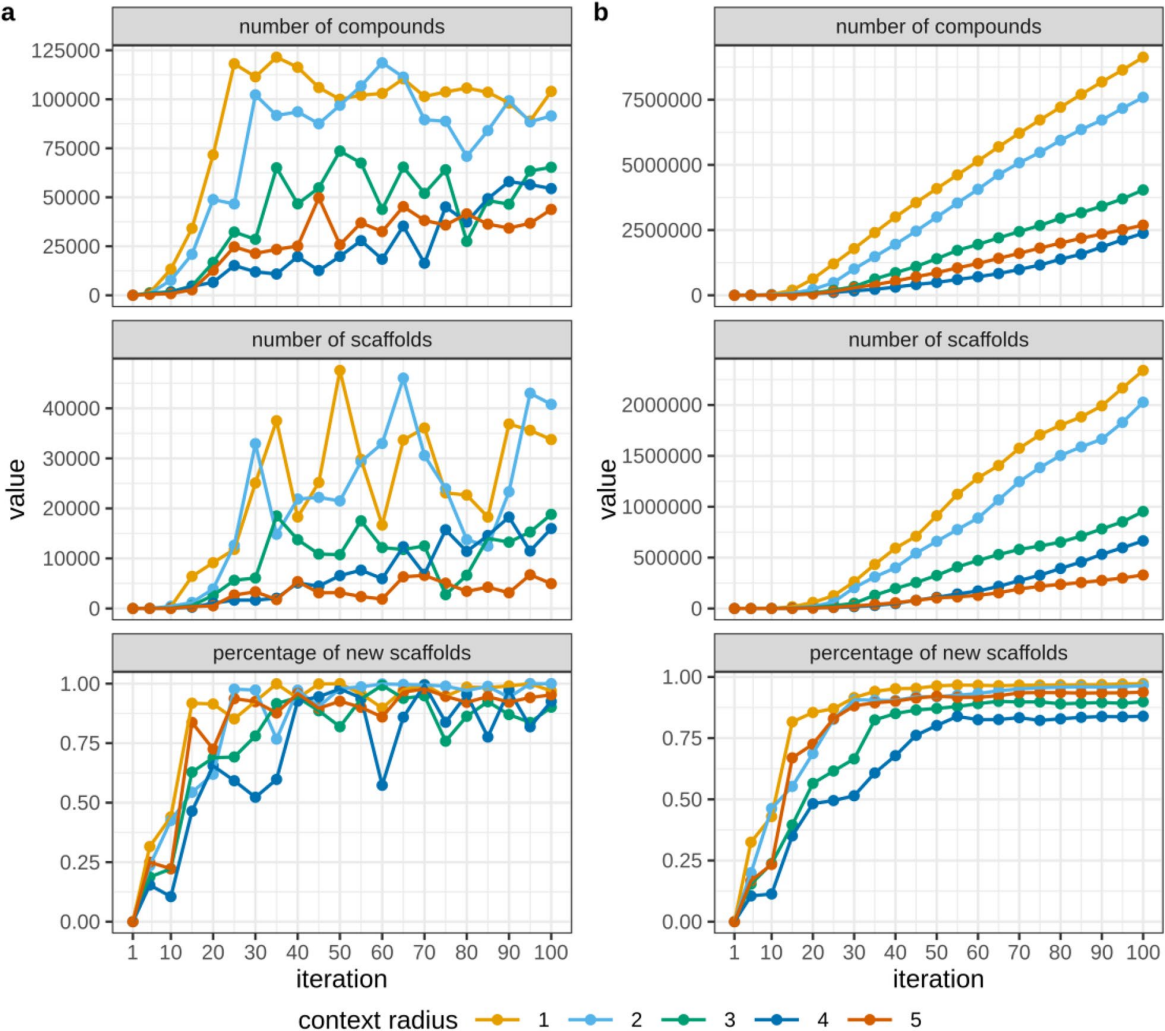
Bemis–Murcko scaffold analysis for compounds generated in course of stochastic exploration of chemical space. **a** Depicts data for each iteration separately. **b** Depicts cumulative statistics over iterations

The stochastic exploration example provides data for analysis of Bemis-Murcko scaffolds to estimate the ability of CReM to generate new scaffolds. Similarly to the previous case study, we observed that the total number of generated compounds decreased with increase in the considered context radius (Fig. 8b). But this number can vary at individual iterations depending on structures selected from a previous iteration (Fig. 8a). The number of distinct scaffolds changed similarly. Their total number increased with increasing the number of iterations but decreased with increasing of the context radius. Enumerated scaffolds were compared with scaffolds in structures used for generation of the fragment database. The percentage of newly generated scaffolds increases quickly. After the twentieth iteration cumulative percentage of novel scaffolds exceeded 50% for all simulations and reached a plateau after 50 iterations. For smaller context radius the percentage of new scaffolds reached 96.5-97.3% (Fig. 8b). The percentage of new scaffolds generated on individual iterations also quickly increased and varied at a reasonably high level (Fig. 8a).

#### Physicochemical properties of stochastically generated compounds

To characterize chemical space accessible with stochastic exploration we analyzed the distribution of different physicochemical properties of generated compounds calculated with RDKit: number of H-bond donors (HBD) and acceptors (HBA), molecular weight (MW), lipophilicity (logP), number of rotatable bonds (RTB), topological surface area (TPSA), number of rings (NumRings), fraction of sp^3^-carbon atoms (Csp3) [44] and fraction of molecular framework (fmf) [45]. We will analyze here the first fifty iterations because distributions were quite robust at later iterations (Additional file 1).

**Fig. 9:**
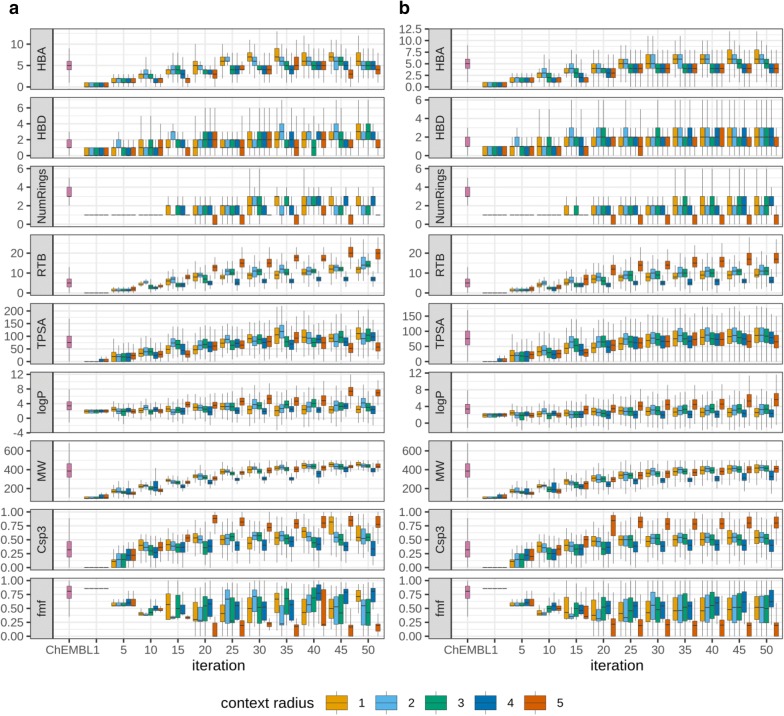
Distributions of physicochemical parameters of compounds generated during stochastic exploration of chemical space in comparison with the same parameters of compounds of the initial ChEMBL data set used for generation of the fragment database

**Fig. 10:**
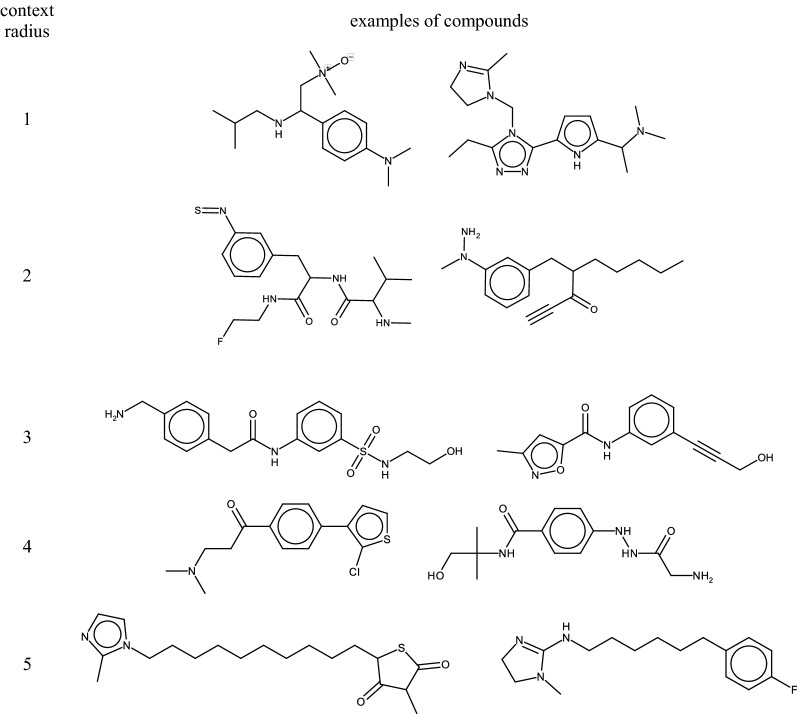
Example of compounds generated at the twentieth iteration at different context radius

The number of H-bond donors and acceptors slowly increased over the iterations and reached values relatively similar to the number of HBD and HBA in compounds from the ChEMBL dataset used for generation of the fragment database (Fig. 9). A similar trend was observed for TPSA values. The number of rings was relatively low over all iterations. The major part of generated compounds contained from 1 to 3 rings whereas compounds containing 3–4 rings were the most frequently occurred in the ChEMBL data set. The distribution of lipophilicity of generated compounds was similar to compounds from ChEMBL and was not changed much during iterations. The only exception was the simulation run with the context of radius 5. In that case, lipophilicity of compounds increased during iterations and reached average logP values greater than 5, whereas for ChEMBL compounds average logP was 3.38. This difference can be easily explained if one will look at the distribution of number of rotatable bonds, fraction of sp^3^-carbons and fraction of molecular frameworks. The very large number of rotatable bonds (17–18) and high fraction of sp^3^-carbons (~ 0.8) in combination with low fraction of molecular framework (~ 0.2) indicated that these compounds contain mostly aliphatic chains. Indeed, after the twentieth iteration, the search will get to chemical space populated with linear aliphatic chains and could not leave it (Fig. 10). But the most striking was the increase of average molecular weight for all simulations irrespective of context radius. Molecular weight was increased over iterations and reached the maximum allowed values close to 500 Da near the 50th iteration. We performed a short fully unrestricted generation and observed that molecular weight constantly grew over iterations and quickly went beyond 500 Da. This can be explained by the fact that the number of fragments in the database grows with the increase in the number of heavy atoms. For the simulation we chose that replacing fragment can have ± 1 heavy atoms relative to the replaced fragment. This, symmetric variance of the size of replacing fragment resulted in the more frequent selection of larger fragments for replacement and, therefore, compounds started to grow.

These results demonstrate that molecular weight or complexity of compounds increases constantly. Lipophilicity, topological surface area, number of H-bond donors and acceptors were more robust and their distribution is more similar to the distribution of fragmented ChEMBL compounds. The number of rings in generated compounds was somewhat lower relative to ChEMBL compounds. Fractions of sp^3^-carbons and molecular framework largely differ from the ChEMBL reference suggesting that more saturated compounds are generated. Despite some properties of compounds being in a similar range relative to reference chemical space, we believe that stochastic sampling of compounds using iterative approaches is not reasonable. A random move in chemical space is performed each iteration and such unguided exploration can go in any direction. If we do not restrict the molecular mass of generated compounds to 500 Da the size of compounds quickly grows, and compounds go beyond this threshold. At the same time, this feature of iterative approaches will be useful if one will want to explore chemical space beyond the reference one.

### Case study 3: Guacamol benchmarks

#### Distribution learning benchmark

To better demonstrate the ability of CReM to reproduce the distribution of reference chemical space we performed several runs of the Guacamol benchmark. A seed compound having molecular weight less than 350 Da was randomly chosen from the reference ChEMBL database. The MUTATE operation was applied to it to enumerate new structures. The size of replaced fragments was set to a range from 0 to 8 heavy atoms. Different size of replacing fragments relative to replaced ones was chosen: ± 2, ± 6, ± 10 and a non-symmetric one from − 10 to 2. The larger difference should result in larger steps and better coverage of chemical space. The maximum number of randomly chosen replacements was set to 2, 5, 10 or 100. A smaller number of replacements should result in more diverse generated compounds and better coverage of chemical space due to the greater number of steps required to generate 10,000 distinct structures. Compounds with molecular weight greater than 500 Da were discarded. A random compound from the generated on a particular iteration was chosen for the next iteration if no compounds with molecular weight less than 500 Da were generated a random compound from an already generated population was picked. Each combination of parameters was tested in three independent runs.

As expected, all generated structures were chemically valid irrespective of the chosen setup (Table 5). Novelty achieved a maximum value in almost all cases. In general the uniqueness of compounds was high. Uniqueness was relatively low and variance was relatively high in cases where the size of replacing fragment was set to ±10, but we cannot explain this behavior. KL divergence was somewhat greater in cases where the larger variation of the size of replacing fragments was allowed and where a smaller number of compounds was selected on each iteration. Moderate KL divergence and low Frechet ChemNet Distance scores showed that CReM could not reproduce the distribution of the reference space well. Thus, this iterative search approach is not very suitable for a stochastic sampling of compounds similar to reference chemical space (Table 5).

**Table 5:** Results for the distribution learning Guacamol benchmarks

Case	Min increase	Max increase	Max replacements	Validity	Uniqueness	Novelty	KL divergence	Frechet ChemNet Distance
CReM	− 2	2	100	1 ± 0	0.935 ± 0.021	1 ± 0	0.443 ± 0.023	0.021 ± 0.007
CReM	− 2	2	10	1 ± 0	0.942 ± 0.008	1 ± 0	0.530 ± 0.061	0.024 ± 0.034
CReM	− 2	2	5	1 ± 0	0.941 ± 0.003	1 ± 0	0.572 ± 0.038	0.044 ± 0.053
CReM	− 2	2	2	1 ± 0	0.950 ± 0.002	1 ± 0	0.551 ± 0.054	0.019 ± 0.018
CReM	− 6	6	100	1 ± 0	0.942 ± 0.023	0.999 ± 0	0.541 ± 0.056	0.018 ± 0.012
CReM	− 6	6	10	1 ± 0	0.924 ± 0.010	1 ± 0	0.603 ± 0.019	0.041 ± 0.045
CReM	− 6	6	5	1 ± 0	0.921 ± 0.022	1 ± 0	0.584 ± 0.034	0.038 ± 0.040
CReM	− 6	6	2	1 ± 0	0.935 ± 0.009	1 ± 0	0.605 ± 0.015	0.053 ± 0.050
CReM	− 10	10	100	1 ± 0	0.918 ± 0.019	1 ± 0	0.531 ± 0.058	0.071 ± 0.027
CReM	− 10	10	10	1 ± 0	0.907 ± 0.022	0.999 ± 0.001	0.622 ± 0.011	0.030 ± 0.016
CReM	− 10	10	5	1 ± 0	0.875 ± 0.025	1 ± 0	0.599 ± 0.035	0.085 ± 0.056
CReM	− 10	10	2	1 ± 0	0.850 ± 0.094	1 ± 0	0.590 ± 0.064	0.006 ± 0.005
CReM	− 10	2	100	1 ± 0	0.945 ± 0.021	0.999 ± 0	0.550 ± 0.037	0.016 ± 0.012
CReM	− 10	2	10	1 ± 0	0.950 ± 0.008	1 ± 0	0.545 ± 0.007	0.045 ± 0.010
CReM	− 10	2	5	1 ± 0	0.956 ± 0.001	1 ± 0	0.533 ± 0.073	0.048 ± 0.036
CReM	− 10	2	2	1 ± 0	0.962 ± 0.006	1 ± 0	0.577 ± 0.027	0.042 ± 0.037
SMILES LSTM^*^				0.959	1	0.912	0.991	0.913
Graph MCTS^*^				1	1	0.994	0.522	0.015
AAE^*^				0.822	1	0.988	0.886	0.529
ORGAN^*^				0.379	0.841	0.687	0.267	0
VAE^*^				0.870	0.999	0.974	0.982	0.963

* Results were taken from the ref [38]

#### Goal-directed benchmarks

We implemented an iterative search algorithm and applied it to all Guacamol goal-directed tasks. If the list of the seed structures was empty the seed structures were chosen randomly from the list of SMILES supplied with the Guacamol and represented the whole ChMEBL database. The size of a population selected on each iteration was set to be equal to the size of the output population, but not less than 10 compounds. To make the search adaptive we adjusted the fragment size of replacement according to the current score of the population. If the score was equal or less than 0.3 (far from the goal) the replacing fragment can differ at most on ± 10 heavy atoms from the replaced one. If the score was greater than 0.8 (close to the goal) the replacing fragment can differ at most on ± 4 heavy atoms from the replaced one. Intermediate fragment sizes (5–9) were chosen if the score was within 0.3–0.8 range. This allows to quickly explore chemical space in the beginning and better tune structures at the end of generation. For each compound in a population up to 1000 randomly chosen mutations were applied. Compounds, which were already used for structure generation, were stored in a separate list and removed from the list of generated structures. Remaining top-scored compounds were selected for the next iteration.

Since the implemented optimization procedure is local and can get stuck in local optima we implemented three levels of “patience”. At the first level if the best score was not improved after three consecutive iterations the fragment size was increased on ± 1 and the number of randomly chosen replacements on 100 irrespectively to the current score. This makes the small stepwise increase in chemical space exploration. If after 10 consecutive iterations no improvement was observed larger changes were applied: the size of replacing fragment was increased on ± 10 and the number of replacements on 500. This would enable the rougher exploration of a chemical space around the best candidates. At the third level, if after 33 iterations no improvement was observed new seed compounds were randomly selected to restart the search but the best found candidates were kept. This procedure was not applied if the seed structure was supplied with the task. The list of already visited compounds was cleared after any change of generator parameters whether this was caused by improving the best score or by exceeding one of “patience” levels.

Some of the target benchmark compounds contain complex ring systems. Therefore, due to the current limitation of the implemented CReM approach to generate new ring systems the whole ChEMBL fragment database was used in this study. The maximum execution time of each task was set to 5 h or maximum of 1000 iterations were allowed.

The results demonstrated that the implemented search algorithm based on CReM approach compared well with the published reference approaches by achieving the highest score in 16 out of 20 tasks (Table 6). However, the total score was slightly lower than the total score of Graph GA approach, which uses the genetic algorithm on molecular graphs. This is mainly due to the considerable advantage demonstrated by Graph GA approach (0.891) over CReM-based approach (0.763) in the task of generation of molecules, which were structurally dissimilar to sitagliptin but had similar lipophilicity and topological polar surface area. Interestingly, the other reference approaches performed even worse in this task. Output results and tuning parameters are available in Additional file 2 and Additional file 3.

**Table 6:** Results for the Guacamol goal-directed benchmarks

Task	SMILES LSTM*	SMILES GA*	Graph GA*	Graph MCTS*	CReM
Celecoxib rediscovery	*1.000*	0.732	*1.000*	0.355	*1.000*
Troglitazone rediscovery	*1.000*	0.515	*1.000*	0.311	*1.000*
Thiothixene rediscovery	*1.000*	0.598	*1.000*	0.311	*1.000*
Aripiprazole similarity	*1.000*	0.834	*1.000*	0.380	*1.000*
Albuterol similarity	*1.000*	0.907	*1.000*	0.749	*1.000*
Mestranol similarity	*1.000*	0.79	*1.000*	0.402	*1.000*
C11H24	*0.993*	0.829	0.971	0.410	0.966
C9H10N2O2PF2Cl	0.879	0.889	*0.982*	0.631	0.940
Median molecules 1	*0.438*	0.334	0.406	0.225	0.371
Median molecules 2	0.422	0.38	0.432	0.170	*0.434*
Osimertinib MPO	0.907	0.886	0.953	0.784	*0.995*
Fexofenadine MPO	0.959	0.931	0.998	0.695	*1.000*
Ranolazine MPO	0.855	0.881	0.92	0.616	*0.969*
Perindopril MPO	0.808	0.661	0.792	0.385	*0.815*
Amlodipine MPO	0.894	0.722	0.894	0.533	*0.902*
Sitagliptin MPO	0.545	0.689	*0.891*	0.458	0.763
Zaleplon MPO	0.669	0.413	0.754	0.488	*0.770*
Valsartan SMARTS	0.978	0.552	0.990	0.04	*0.994*
Deco Hop	0.996	0.970	*1.000*	0.590	*1.000*
Scaffold Hop	0.998	0.885	*1.000*	0.478	*1.000*
Total score	17.341	14.398	*17.983*	9.011	17.919

Italic signifies the highest achieved value for a particular task (row)

* Results were taken from the ref [38]

## Conclusion

The developed CReM approach of structural transformations generates chemically valid structures by design. This approach also enables one to indirectly influence generation outcome by customizing an input compound database, which is then used for generation of a database of interchangeable fragments. The selection of more synthetically accessible input compounds for fragmentation can result in more synthetically accessible generated compounds. The performed experiments showed that even with a small library of synthetically feasible compounds (27,916 ChEMBL compounds with SCscore ≤ 2) one may generate rather diverse sets of structures with better predicted synthetic feasibility. However, there is always a trade-off between the size of a fragment database and novelty and diversity of generated structures. An end-user can also choose a more conservative generation strategy by increasing the value of the considered context radius. This avoids generation of new structural motifs with size lesser than the chosen radius value. A combination of a custom input compound database and chosen context radius gives flexible control over the chemotypes of generated structures, their number, diversity, and synthetic feasibility.

We also demonstrated that iterative search strategies based on CReM approach are more suitable for generation of compounds with particular properties and less suitable for a stochastic sampling of compounds similar to ones used for generation of a fragment database.

The major limitation of the current implementation is the inability to create new ring systems. Therefore, the diversity of ring systems in generated compounds completely depends on their representativeness in the input compound database. The developed framework can be used in combination with any modeling tools to create custom workflows for chemical space exploration and optimization of compound properties.

## Supplementary information

**Additional file 1.** The lists of PAINS patterns found in generated structures based on the PAINS-less ChEMBL fragment database and distributions of physicochemical properties of stochastically generated compounds.

**Additional file 2.** Output of the Guacamol goal-directed benchmarks.

**Additional file 3.** Tuning parameters used to generate compounds based on CReM framework for the Guacamol goal-directed benchmarks.

## Data Availability

Project name: CReM Project home page: http://www.qsar4u.com/pages/crem.php GitHub: https://github.com/DrrDom/crem Operating system(s): cross-platform Programming language: Python 3 Other requirements: RDKit 2017.09 or higher License: BSD 3-clause Any restrictions to use by non-academics: no.
