# Supplementary material for: CReM: chemically reasonable mutations framework for structure generation
Source: J Cheminform. 2020 Apr 22;12:28. doi: 10.1186/s13321-020-00431-w (PMC7178718; doi:10.1186/s13321-020-00431-w)
Supplement: Supplementary file 1 — Additional file 1. The lists of PAINS patterns found in generated structures based on the PAINS-less ChEMBL fragment database and distributions of physicochemical properties of stochastically generated compounds. [file 13321_2020_431_MOESM1_ESM.pdf]

CReM: chemically reasonable mutations framework for structure generation

Pavel Polishchuk

Table S1. PAINS patterns found in stochastically generated compounds using the PAINS-less ChEMBL fragment database and context radius 1.

	Number of compounds	PAINS pattern
1.	10	anil_alk_C(1)
2.	1	anil_alk_D(1)
3.	626001	anil_di_alk_A(478)
4.	31554	anil_di_alk_B(251)
5.	34928	anil_di_alk_C(246)
6.	332531	anil_di_alk_D(198)
7.	743604	anil_di_alk_E(186)
8.	155	anil_di_alk_F(14)
9.	4544	anil_di_alk_G(9)
10.	2	anil_di_alk_I(4)
11.	4	anil_di_alk_J(3)
12.	20	anil_di_alk_K(2)
13.	1309	anil_NH_alk_B(3)
14.	43	anil_NH_alk_C(2)
15.	4	anil_NH_alk_D(2)
16.	19	anil_no_alk(40)
17.	1	anil_OC_alk_D(2)
18.	2	anil_OC_alk_E(1)
19.	2	anil_OC_alk_F(1)
20.	3	anil_OC_no_alk_A(8)
21.	4	anil_OC_no_alk_C(3)
22.	23	anisol_A(5)
23.	22	anisol_B(2)
24.	3	anthranil_acid_A(19)
25.	1	anthranil_acid_G(1)
26.	350	anthranil_one_A(38)
27.	17	catechol_A(92)
28.	1	cyanamide_A(1)
29.	5	cyano_cyano_B(3)
30.	2	cyano_ene_amine_C(3)
31.	1	cyano_imine_B(17)
32.	263	cyano_keto_A(2)
33.	10	dhp_keto_A(9)
34.	2	diazox_A(3)
35.	1170	dyes5A(27)
36.	66	ene_cyano_A(19)

37.	6	ene_cyano_C(6)
38.	259	ene_cyano_E(1)
39.	1	ene_cyano_G(1)
40.	2	ene_one_B(2)
41.	60	ene_one_D(1)
42.	45	ene_one_ester(24)
43.	662	ene_one_hal(17)
44.	72	ene_one_yne_A(1)
45.	2	ene_rhod_C(13)
46.	4	furan_acid_A(4)
47.	2	het_5_B(4)
48.	99	het_thio_N_5B(2)
49.	2	het_thio_N_5C(1)
50.	87	hzone_anil_di_alk(35)
51.	163	hzone_enamin(30)
52.	1	hzone_furan_A(6)
53.	261	hzone_phenol_A(479)
54.	21	hzone_phenol_B(215)
55.	3	hzone_pyrrol(64)
56.	4	hzone_thiophene_B(4)
57.	4	imidazole_B(2)
58.	6	imine_ene_A(5)
59.	13771	imine_one_A(321)
60.	5	imine_one_B(4)
61.	184	imine_phenol_A(3)
62.	22	indol_3yl_alk(461)
63.	2	keto_keto_beta_D(5)
64.	5509	mannich_A(296)
65.	262	pyrrole_A(118)
66.	5	pyrrole_B(29)
67.	15	pyrrole_C(8)
68.	1	pyrrole_D(5)
69.	27	pyrrole_E(5)
70.	1	pyrrole_G(4)
71.	16	pyrrole_L(1)
72.	3	pyrrole_M(1)
73.	23	pyrrole_N(1)
74.	17	pyrrole_O(1)
75.	3	rhod_sat_C(3)
76.	48	sulfonamide_B(41)
77.	3	sulfonamide_E(2)
78.	3	sulfonamide_F(1)
79.	7	thiaz_ene_B(17)
80.	92	thiazole_amine_A(4)
81.	1	thiazole_amine_G(2)
82.	247	thiazol_SC_A(3)

83.	5	thio_amide_C(2)
84.	39	thio_amide_D(2)
85.	1	thio_amide_E(1)
86.	7	thio_amide_F(1)
87.	84	thio_carbam_A(1)
88.	40	thio_ester_B(4)
89.	1	thio_keto_het(2)
90.	5889	thio_ketone(43)
91.	4	thiophene_amino_Ab(40)
92.	13	thiophene_amino_B(12)
93.	1	thiophene_amino_D(3)
94.	2	thiophene_amino_G(2)
95.	15	thiophene_amino_H(2)
96.	1	thiophene_D(2)
97.	1	thiophene_F(1)
98.	6	thiophene_hydroxy(28)
99.	2	thio_urea_C(9)
100.	2	thio_urea_D(8)
101.	1	thio_urea_G(5)
102.	2	thio_urea_L(1)

Table S2. PAINS patterns found in stochastically generated compounds using the PAINS-less ChEMBL fragment database and context radius 2.

	Number of compounds	PAINS pattern
1.	7	anil_alk_D(1)
2.	316	anil_di_alk_A(478)
3.	639	anil_di_alk_B(251)
4.	27928	anil_di_alk_C(246)
5.	863	anil_di_alk_D(198)
6.	343	anil_di_alk_E(186)
7.	3	anil_di_alk_F(14)
8.	2	anil_di_alk_I(4)
9.	21	anil_NH_alk_B(3)
10.	11	anil_no_alk(40)
11.	2	anil_OC_alk_E(1)
12.	2	anil_OH_alk_A(8)
13.	1	anisol_A(5)
14.	32	anisol_B(2)
15.	13	anthranil_one_A(38)
16.	11	catechol_A(92)
17.	1	cyano_ene_amine_C(3)
18.	5	cyano_keto_A(2)
19.	1	diazox_A(3)
20.	245	dyes5A(27)
21.	10	ene_cyano_A(19)
22.	3	ene_cyano_E(1)
23.	2	ene_one_D(1)
24.	25	ene_one_hal(17)
25.	10	ene_rhod_B(16)
26.	2	het_55_A(2)
27.	1	hzone_enamin(30)
28.	26	hzone_phenol_A(479)
29.	84	hzone_phenol_B(215)
30.	7	hzone_pipzn(79)
31.	4	hzone_thiophene_B(4)
32.	5	imine_one_A(321)
33.	10	imine_phenol_A(3)
34.	135	mannich_A(296)
35.	100	pyrrole_A(118)
36.	3	pyrrole_D(5)
37.	2	pyrrole_E(5)
38.	3	pyrrole_N(1)
39.	6	sulfonamide_B(41)
40.	17	tetrazole_A(1)
41.	4	thiaz_ene_B(17)
42.	19	thiazole_amine_B(3)
43.	26	thiazole_amine_L(1)

44.	66	thio_carbam_A(1)
45.	2	thio_ester_B(4)
46.	2	thio_ketone(43)
47.	13	thiophene_amino_Ab(40)
48.	13	thiophene_amino_G(2)
49.	4	thiophene_D(2)
50.	1	thiophene_E(2)
51.	25	thiophene_hydroxy(28)
52.	1	thio_thiomorph_Z(1)

Table S3. PAINS patterns found in stochastically generated compounds using the PAINS-less ChEMBL fragment database and context radius 3.

	Number of compounds	PAINS pattern
1.	767	anil_di_alk_A(478)
2.	181	anil_di_alk_B(251)
3.	214474	anil_di_alk_C(246)
4.	656	anil_di_alk_D(198)
5.	1305	anil_di_alk_E(186)
6.	5	anil_di_alk_F(14)
7.	32	anil_di_alk_G(9)
8.	38	anil_di_alk_I(4)
9.	171	anil_NH_alk_B(3)
10.	36	anil_no_alk(40)
11.	2	anil_OC_no_alk_A(8)
12.	3	anil_OH_alk_A(8)
13.	529	dyes5A(27)
14.	73	ene_cyano_A(19)
15.	1	ene_rhod_C(13)
16.	8	het_thio_5_A(8)
17.	51	het_thio_5_C(2)
18.	2	hzone_phenol_A(479)
19.	1	hzone_phenol_B(215)
20.	4	hzone_phenone(7)
21.	11	imine_one_A(321)
22.	185	mannich_A(296)
23.	96	pyrrole_A(118)
24.	2	pyrrole_D(5)
25.	4	pyrrole_G(4)
26.	3	thiazole_amine_B(3)
27.	1	thio_urea_D(8)
28.	5	thio_urea_E(7)

Table S4. PAINS patterns found in stochastically generated compounds using the PAINS-less ChEMBL fragment database and context radius 4.

	Number of compounds	PAINS pattern
1.	1	anil_alk_B(1)
2.	3	anil_alk_bim(9)
3.	13	anil_alk_D(1)
4.	1249	anil_di_alk_A(478)
5.	615	anil_di_alk_B(251)
6.	4887	anil_di_alk_C(246)
7.	10556	anil_di_alk_D(198)
8.	12476	anil_di_alk_E(186)
9.	2	anil_di_alk_F(14)
10.	66	anil_di_alk_G(9)
11.	59	anil_NH_alk_B(3)
12.	5	anil_NH_alk_D(2)
13.	111	anil_no_alk(40)
14.	9	anisol_B(2)
15.	1	ene_rhod_B(16)
16.	1	het_thio_5_C(2)
17.	1	hzone_anil(14)
18.	2	hzone_phenol_A(479)
19.	1	hzone_phenol_B(215)
20.	3	hzone_phenone(7)
21.	1	mannich_A(296)
22.	4	misc_aminoacid_A(1)
23.	1	pyrrole_G(4)
24.	120	thiazole_amine_B(3)
25.	1	thiazol_SC_A(3)
26.	1	thio_urea_B(9)

Table S5. PAINS patterns found in stochastically generated compounds using the PAINS-less ChEMBL fragment database and context radius 5.

	Number of compounds	PAINS pattern
1.	29	anil_di_alk_C(246)

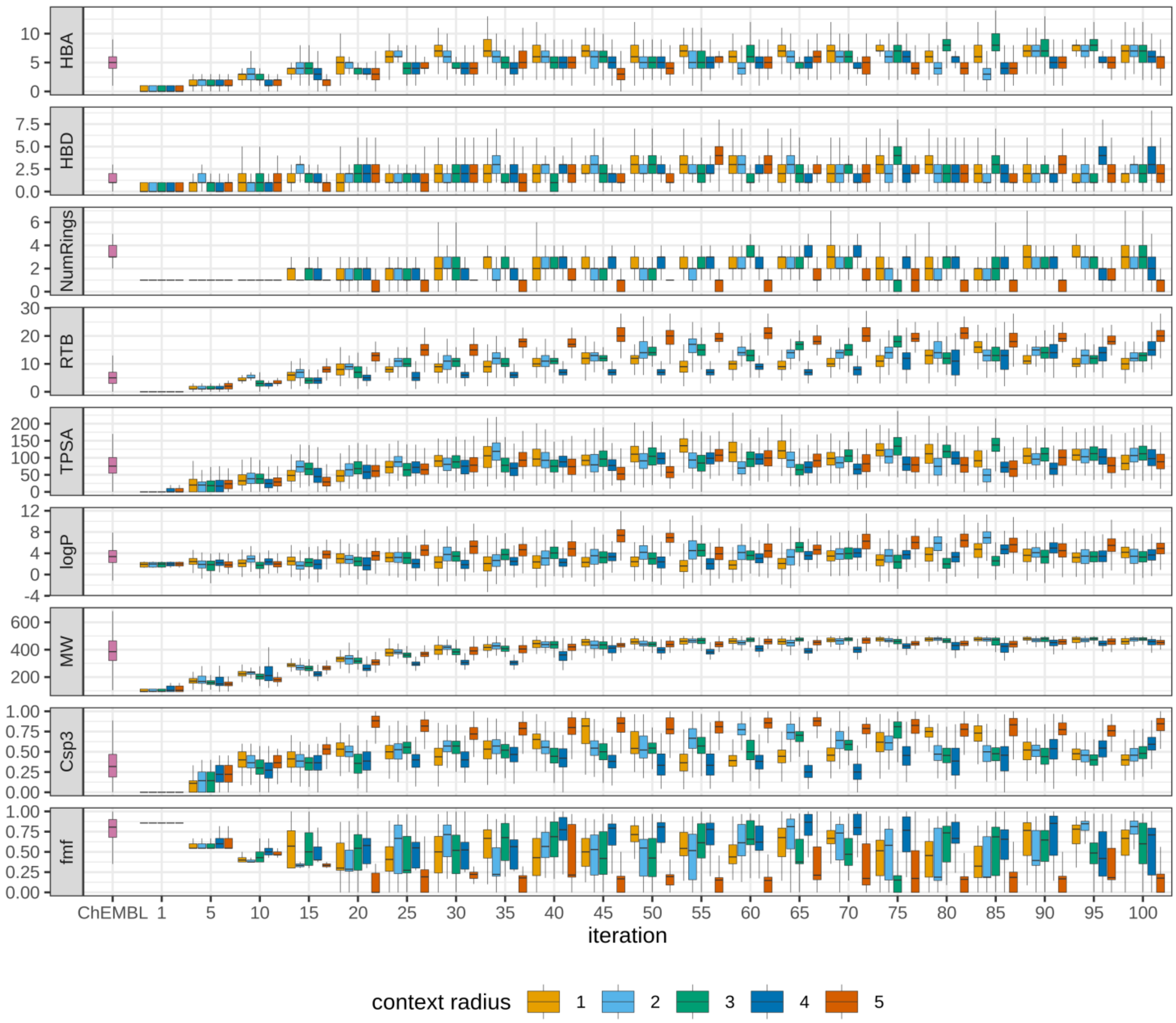

Fugure S1. Distributions of physicochemical parameters of compounds generated during stochastic exploration of chemical space at specific iterations in comparison with the same parameters of compounds of the initial ChEMBL data set used for generation of the fragment database.

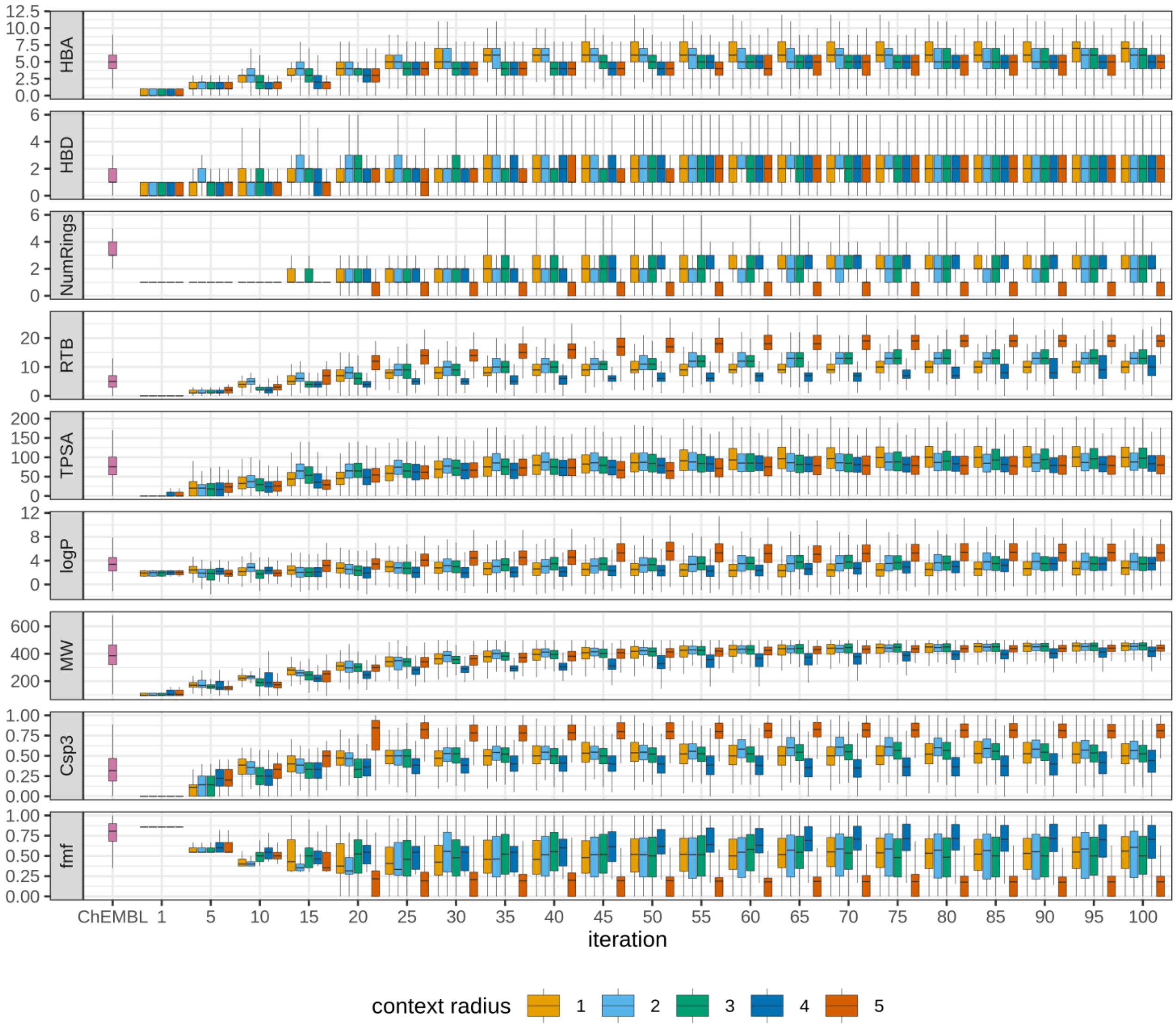

Figure S2. Cumulative distributions of physicochemical parameters of compounds generated during stochastic exploration of chemical space in comparison with the same parameters of compounds of the initial ChEMBL data set used for generation of the fragment database.
